# A case report of multiple extramedullary plasmacytoma of the head and neck

**DOI:** 10.1097/MD.0000000000032203

**Published:** 2022-12-02

**Authors:** Jin Zhang, Detao Ding, Juxing Sun, Hui Zhang, YunBing Dai, Xiaoying Li, Xu Ma, Xiaoyu Li, Yungang Wu

**Affiliations:** a School of Clinical Medicine Jining Medical University, Jining, Shandong, China; b Department of Otolaryngology-Head and Neck Surgery, The Affiliated Hospital of Jining Medical University, Jining, Shandong, China.

**Keywords:** extramedullary plasmacytoma, multiple, plasma, surgery

## Abstract

Multiple extramedullary plasmacytoma (EMP) of the head and neck is rare and a definitive first-line treatment has not been established. EMP successively as a benign mass occurring in multiple areas of the head and neck is rare. This report describes an atypical case with EMP occurring in multiple areas of the head and neck.

**Patient concerns:**

A 35-year-old woman was admitted due to complaints of sore throat discomfort accompanied by hoarseness. The patient had undergone surgical excision of the thyroid gland and parotid gland excision several years ago. Postoperative pathological examination both indicated EMP. This time, the woman suffered EMP in head and neck who was treated with a simple surgery.

**Diagnosis:**

Postoperative pathological examination of the tumor indicated EMP, and histopathological findings revealed the tumor to be a plasmacytoma. Immunopathological examination were consistent with the diagnosis of EMP.

**Interventions:**

The patient underwent surgical resection without radiotherapy.

**Outcomes:**

Histopathological and immunopathological examination findings revealed the tumor to be EMP. The patient was recurrence-free and did not progress to multiple myeloma (MM) during 19 months follow-up.

**Lessons:**

Increasing the awareness of EMP of head and neck is warranted. Our case confirmed that surgical excision is beneficial in the treatment of small, localized EMP.

## 1. Introduction

Plasmacytomas are essentially characterized by abnormal monoclonal proliferation of mature B-cells, producing monoclonal globulin by clonal expansion. Extramedullary plasmacytoma (EMP) is an uncommon plasma cell neoplasm.^[1]^ Burgos-Blasco et al^[2]^ suggested that EMP accounts for only 3% of all plasma cell neoplasms. Christoph et al^[3]^ revealed that EMP belonging to the category of non-Hodgkin lymphoma is a rare entity. EMPs account for 4% of all plasma cell tumors. Guowei Li et al^[4]^ showed that EMP makes up about 5% of all plasmacytomas. EMP reveals a favorable prognosis compared with other plasma cell dyscrasia, such as solitary plasmacytoma of bone and multiple myeloma (MM). Most cases of EMP are seen in older men (male: female ratio of 3:1), with a peak incidence in the 50 to 60-year-old group,^[5]^ with no racial predilection.^[6]^ EMP patients characteristically present with localized disease, and the incidence of lymph node involvement fluctuates around 10% to 20%.^[7]^ Owing to the rarity of EMP, most studies had been retrospective series and case reports, with no randomized trials. Experience in the diagnosis and treatment of EMP is limited, and EMP of the larynx has not been thoroughly studied. The multiplicity has been rarely presented. To provide references for clinical therapy, we describe the rare case of EMP arising from the larynx.

## 2. Case presentation

### 2.1. Clinical history

**Figure 1.**
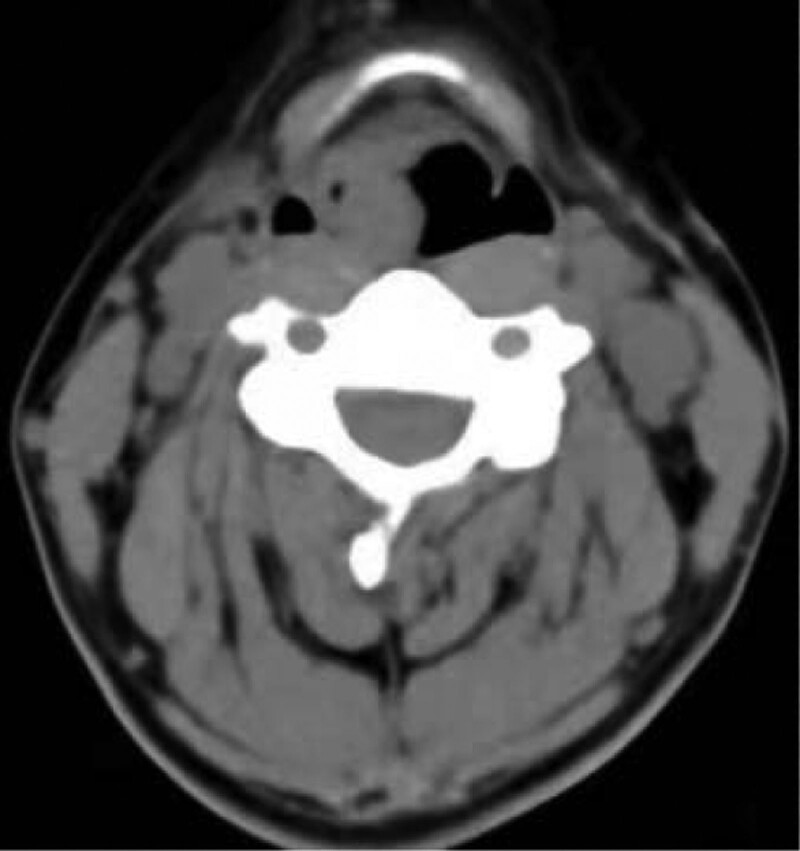
The CT imaging data of the patient. CT = computed tomography.

**Figures 2.**
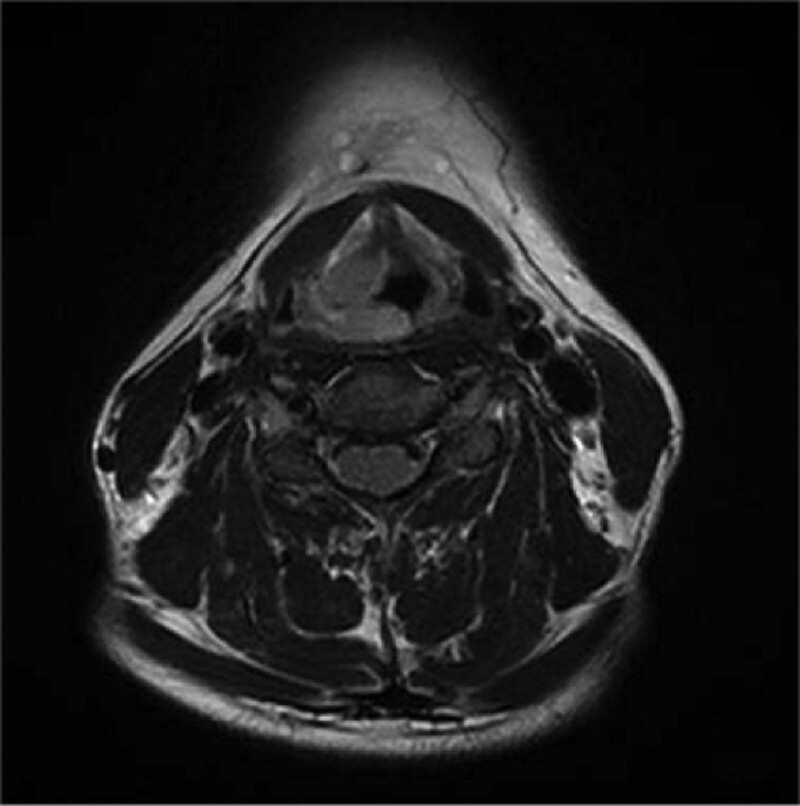
Head and Neck MRI: Head and Neck MRI revealed that the contrast-enhancing soft tissue is located in the posterior wall of the nasopharynx, the right wall of the oropharynx, the right arytenoepiglottic fold, and the right ventricular zone thickened irregularly with axial section. MRI = magnetic resonance imaging.

**Figure 3.**
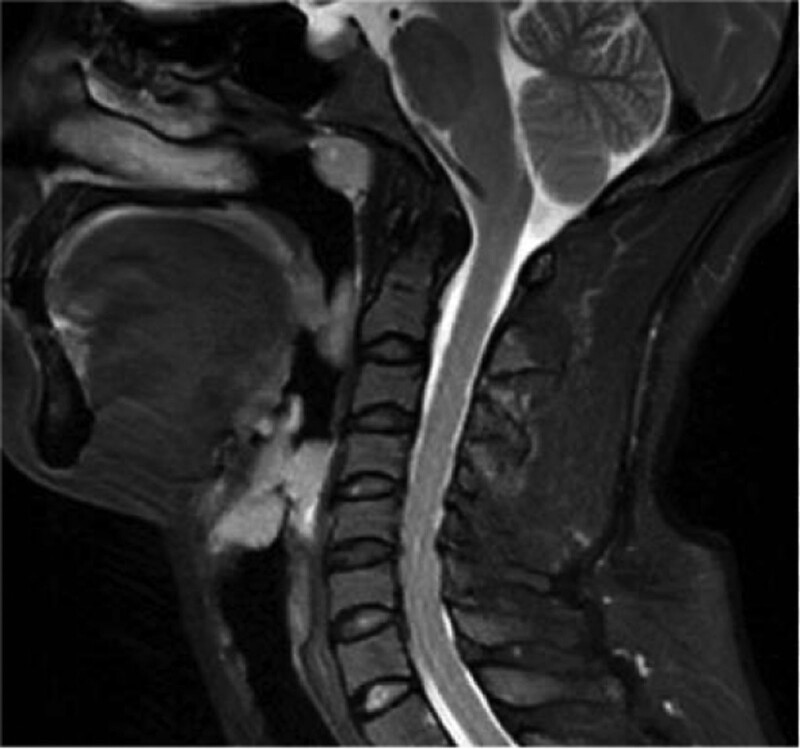
Head and Neck MRI of the sagittal section.

**Figure 4.**
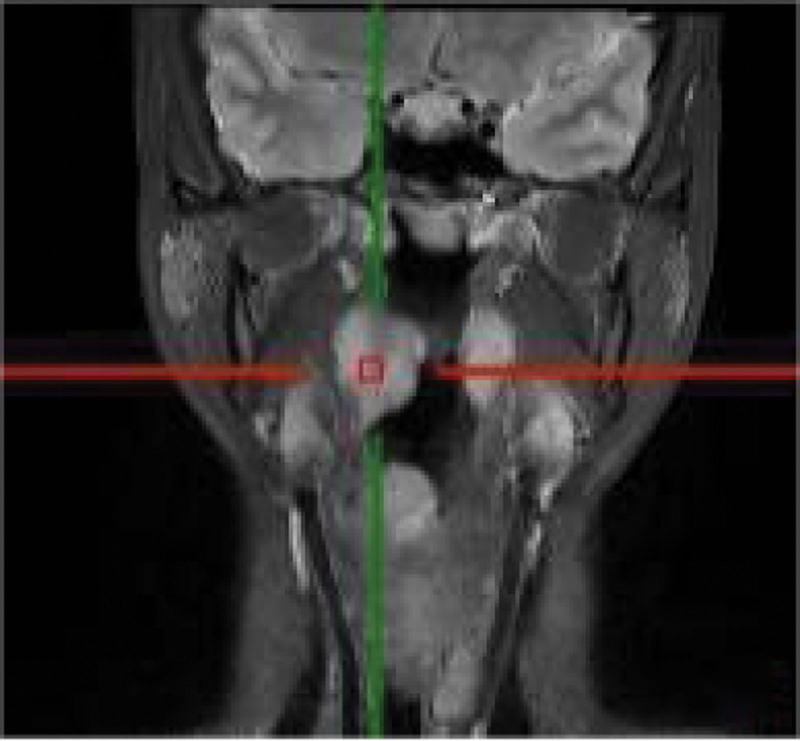
Head and Neck MRI of the coronal section.

**Figure 5.**
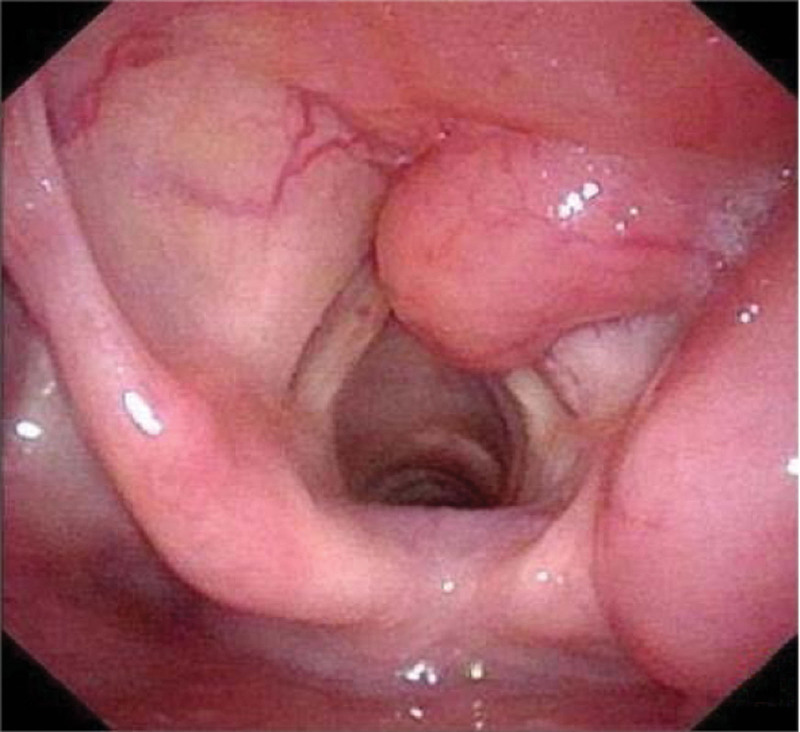
An electronic video laryngoscope revealed mass localized in the aryepiglottic fold and ventricular zone.

**Figures 6.**
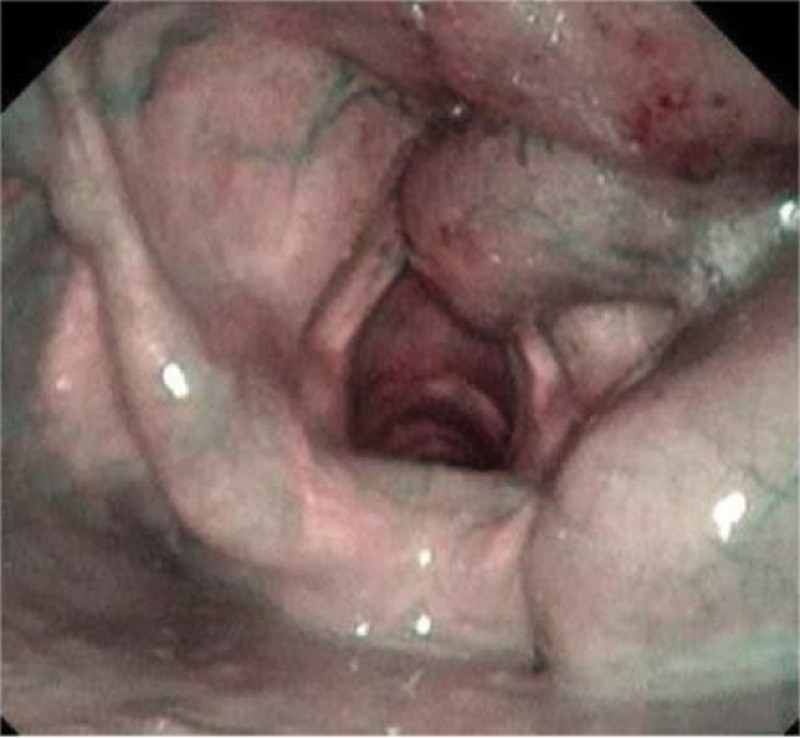
The larynx obtained by Narrow-Band Imaging of electronic video laryngoscope.

**Figure 7.**
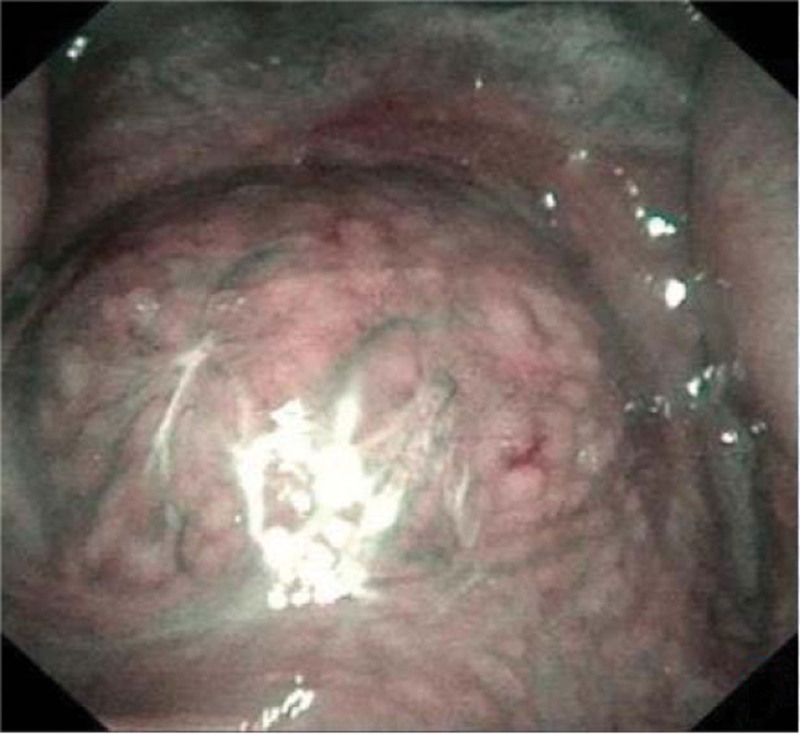
The nasopharyngeal obtained by Narrow-Band Imaging of electronic video laryngoscope.

**Figure 8.**
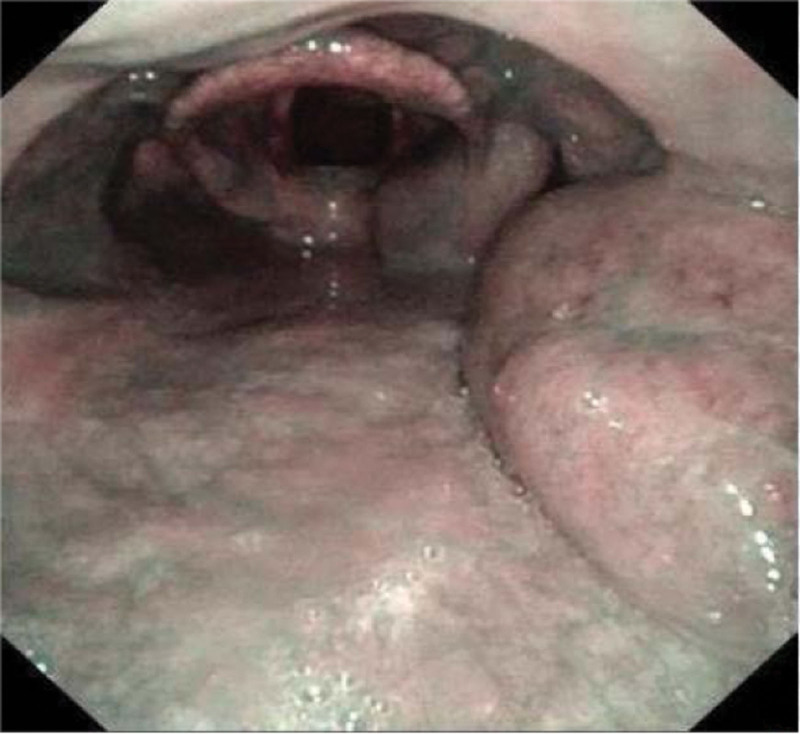
An electronic laryngoscope revealed mass localized in the nasopharynx.

A 35-years-old female patient suffering from sore throat discomfort accompanied by hoarseness lasting for a month was diagnosed with a laryngeal tumor in February 2021. The patient did not present dysphagia or dyspnea, and she had never smoked in her life. Her medical history did reveal an abnormality. The particularity is that she had undergone surgical excision of the thyroid gland in another institution and excision of the parotid gland in stomatology department of our hospital after several months. Postoperative pathological examination both indicated EMP. The patient was admitted with swelling in the left parotid area and had a fever. The palpable mass in the left parotid gland area was 3.0 cm × 2.0 cm × 2.0 cm in size, with no tenderness. Ultrasonography revealed a solid lesion on the left parotid gland, and multiple swollen lymph nodes were detected bilaterally on both parotid glands. A biopsy was performed before surgery, and the mass was revealed to be benign lesions of the salivary glands or inflammation. The patient underwent an excision of the parotid gland mass and facial nerve dissection. Intraoperative rapid pathological examination of the lesion indicated lymphoproliferative lesions, and the postoperative pathological examination consultation results indicated plasmacytoma. Immunopathological examination revealed that CD56 lambda was negative and that CD138 kappa was positive. No neck lymph nodes enlargement was palpable. Electronic video laryngoscopy revealed a mass with a smooth surface and normal mucosal color in the nasopharynx, the right wall of the oropharynx, aryepiglottic fold, and ventricular zone (Fig. 5). The Narrow-Band Imaging showed no signs of malignancy (Fig. 6–8). Head and Neck computed tomography (CT) (Fig. 1) showed: nodular soft tissue density shadow protruding into the cavity with smooth edges in the right arytenoepiglottic-ventricular bands and had no bone or cartilage involvement. The soft tissue of the right oropharynx and the nasopharynx was thicker. The right lobe of the thyroid was absent. Moreover, no swollen lymph nodes were detected bilaterally on both sides. Head and Neck magnetic resonance imaging (MRI) (Fig. 2–4) revealed that the contrast-enhancing soft tissue located in the posterior wall of the nasopharynx, the right wall of the oropharynx, the right arytenoepiglottic fold, and the right ventricular zone thickened irregularly, being isointense on T1 and hyperintense on T2. Diffusion weight imaging revealed restricted diffusion, and there was no recurrence of thyroid and parotid masses in CT and MRI. Laboratory examinations, including complete blood cell count, urinalysis, and abdominal ultrasonography, were normal. A biopsy was performed prior to surgery. Nevertheless, a biopsy of the mass was performed under local anesthesia, and microscopic observation detected chronic inflammation of the mucosa with lymphadenia.

### 2.2. Treatment

**Figure 9.**
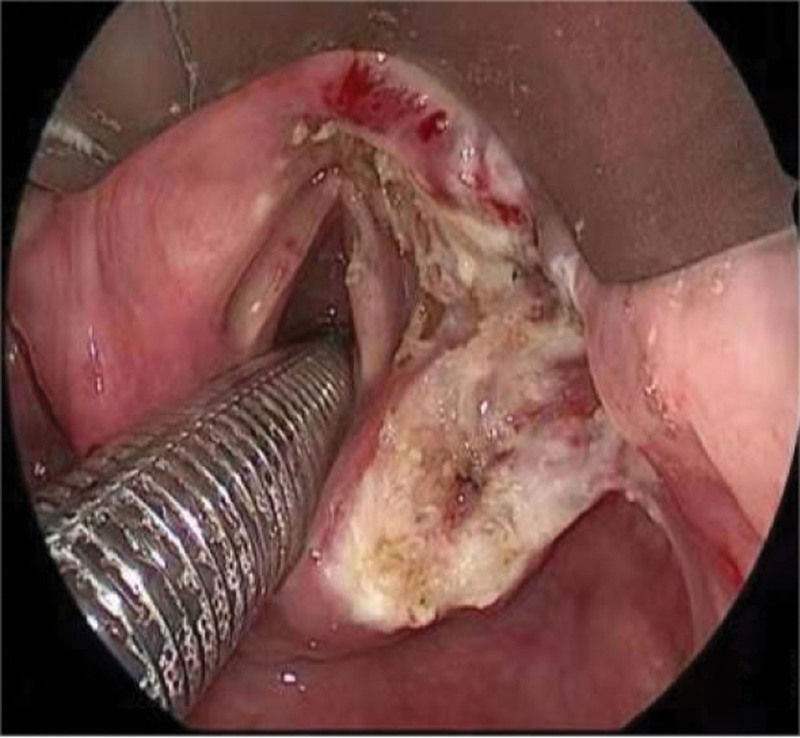
Intraoperative supportive laryngoscopy showed that the mass was in the right lateral laryngeal wall.

The patient underwent a resection of the tumor with a suspension laryngoscope, with no administration of further therapy. The mass was excised with a plasma cutting machine (Fig. 9). The mass was located in the aryepiglottic fold and ventricle zone with a laryngoscope. The oropharynx was exposed by mouth gap, and the mass was found between the right tonsil and the posterior wall of the pharynx, with a size of 1.5 × 2.0 cm. Moreover, we found that the mass arising from the nasopharynx was about 2.0 × 2.0 cm with a 70° endoscope. All the masses were excised at about 3 mm along their periphery. The mass was gray and slightly harder than the surrounding tissues. The bleeding was completely stopped, and the absence of no residual masses was confirmed. Intraoperative rapid pathological examination of the lesion suggested that lymphoproliferative lesions do not exclude lymphoma.

### 2.3. Pathology and diagnosis

**Figure 10.**
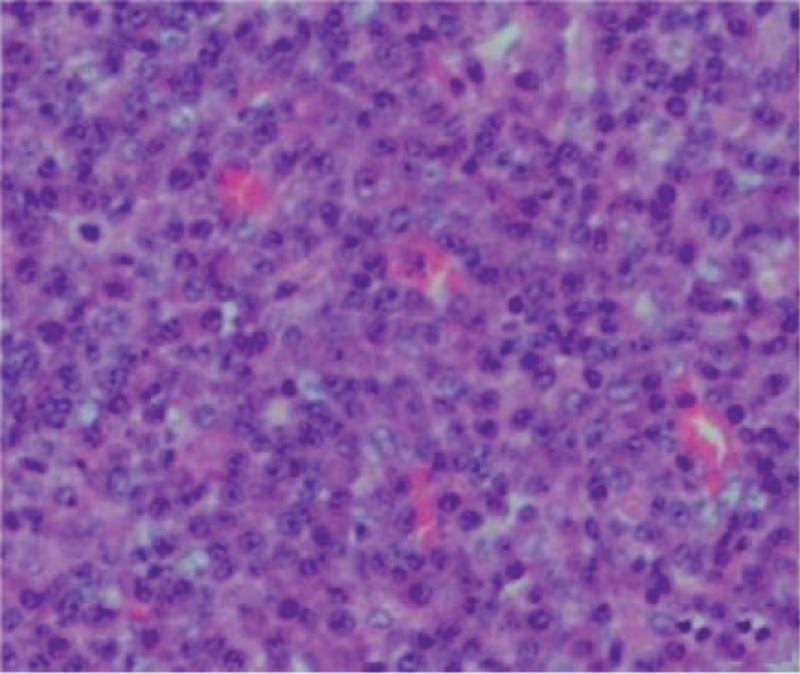
Postoperative pathological examination of the tumor indicated multiple plasma cell infiltration.

Postoperative pathological examination of the tumor indicated EMP, and histopathological findings revealed the tumor to be a plasmacytoma (Fig.10). Immunopathological examination revealed that CD20, CD56, EBER, CyclinD1, and lambda were negative and that CD38, CD138, CD79a, Mum-1, and kappa were positive, thereby leading to EMP diagnosis.

### 2.4. Outcome

**Figure 11.**
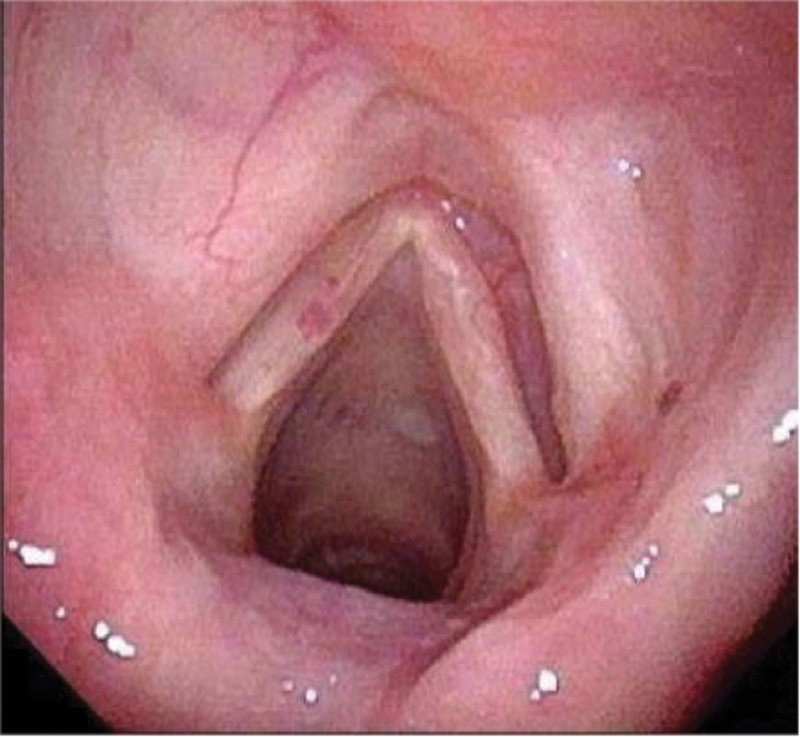
The larynx obtained by an electronic video laryngoscope in 9 months postoperatively.

After follow-up for 20 months, the patient was in good condition following surgery. The thyroid and parotid gland tumor had been excised for 6.5 years. After surgery, the patient underwent further investigations. The tumor was recurrence-free and did not progress to MM during follow-up. Bone marrow aspiration performed at another hospital revealed to be normal with no evidence of tumor deposits. A renal function panel, a complete blood count, electrolyte, and calcium measurements, were all within normal limits. Moreover, the laryngoscope showed normal postoperative changes (Fig. 11).

## 3. Discussion

A plasma cell tumor is a malignant tumor caused by abnormal proliferation of the plasma cell system. Plasma cell neoplasms entail several chronic diseases, such as solitary plasmacytoma of bone, EMP, and MM. EMP occurs outside the bone marrow and can appear in any organ system.^[8]^ Our patient successively found EMP masses in the thyroid gland, parotid gland, larynx, nasopharynx, and lateral pharyngeal wall, which is a rare occurrence. However, the patient’s first thyroidectomy was performed in another hospital, and we remain unaware of their management protocols. In both of the surgeries performed in our hospital, preoperative biopsies were used to confirm the diagnosis of EMP, and the results of postoperative cases were obtained through outpatient consultation. The patient did not undergo radiotherapy or chemotherapy on the 3 occasions, and the tumors did not invade the surrounding bone and cartilage.

Although the clinical presentation varies according to the involved organ^[9]^ and tumor size, EMP of the larynx often presents with hoarseness or dysphagia, without special clinical manifestations,^[10]^ due to the non-specificity in the clinical symptoms, laryngoscope findings, and imaging examinations, it is easily misdiagnosed. The diagnosis of EMP is based on the exclusion of systemic plasma cell proliferative disorders and immunohistochemistry results. Histological diagnosis of EMP requires the presence of clonal plasma cells. Immunohistochemical markers include CD79a, CD38, and CD138, and monoclonal cytoplasmic lambda or kappa light chain expression.^[1]^ José Antônio Pinto et al^[6]^ suggested that imaging tests rule out lytic lesions. MRI is the test of choice to highlight the lytic lesions. CT usually demonstrates a well-defined or infiltrative soft tissue mass, appearing with a mild-to-moderate contrast enhancement. The MRI scans of most EMPs is iso/hypointense to skeletal muscle on T1 and iso/hyperintense on T2-weighted image. Diffusion weight imaging demonstrates restricted diffusion, indicating high cellularity of the lesions.^[11]^ Caers J et al demonstrated that CT might be helpful for an adequate loco-regional staging to EMP as regional lymph node recurrences occur in 7% of EMP cases. It can help with the observation of the size and location of the mass and determine the site of tumor invasion. Although the sensitivity of MRI to detect lytic bone lesions is lower than that of CT, MRI can detect soft tissue. The use of diffusion-weighted MRI, which is a new, highly sensitive technique to detect and monitor tumor lesions in the soft tissues, could improve the ability of MRI to discern between active and inactive lesions. Caers J et al recommended that in addition to the skeletal survey or CT, MRI or PET-CT scans are supposed to exclude the presence of additional lesions, and the use of at least one of these examinations is mandatory. Salaun et al reported that PET-CT outperforms MRI in the diagnosis and follow-up of plasmacytoma patients, although this study was performed in the context of extramedullary spread during MM.^[12]^ Although, over the past decade, PET-CT has increasingly been used in the prognosis of MM; however, its routine use is still hampered by several factors, including high cost, disparities in insurance policies between regions, and limited availability. Ryu SW et al^[13]^ suggested that the use of ultrasonography is limited by the location of the plasmacytoma, such as in the abdomen, breast, subcutaneous tissue, and skeletal muscles. In our case, we performed CT, MRI, and biopsy before surgery, and no malignant tendencies were noted. Therefore, we did not perform bone marrow puncture or radiation therapy after surgery.

The optimal therapy of EMP remains controversial. Due to the rarity of EMP, most studies had been retrospective series and case reports. It is generally considered that treatment strategies included surgical excision, systemic chemotherapy, radiotherapy, or a combination of those.^[13]^ Ge et al^[14]^ suggested that surgical excision, either alone or with adjuvant radiotherapy, has been proposed and proved to be more beneficial than radiotherapy alone. Takahiro et al^[15]^ showed that surgical excision offers better survival outcomes in the therapy of small and localized EMP of the larynx. Alexiou et al^[3]^ showed that surgery suffices provided the mass is localized and resectable. We did not find any cases of successive EMP of the head and neck during the literature review. The prognosis of patients is no recurrence or progression of MM. However, imaging investigations in our patient did not reveal an aggressive and invasive tumor. On the contrary, they were isolated and were surgically excised without radiotherapy. No local recurrence or deterioration has been observed since the first surgery. Hence, the treatment regimen should be devised with caution, and laryngeal function should be preserved.

EMP is a localized entity. However, in 16% of the cases, the EMP can deteriorate to MM, and the progression usually affects the prognosis. Therefore, the diagnosis of EMP is based on the exclusion of MM. Systemic examinations rule out bone involvement. In addition to tumor biopsies, comprehensive laboratory investigations including complete blood cell count, urinalysis, abdominal ultrasonography, bone X-ray examination, immunoglobulin test, and bone marrow cytology should be performed. EMP has good prognosis, Glasbey et al^[1]^ suggested that the 5-year local overall survival rates are between 78.4% and 87.4% for EMP. Guowei Lu et al^[3]^ indicated that cumulative 5-year survival rates are 80%, but about 20% of patients of EMP are prone to recurrence, and 15% of patients can progress to MM. Kamijo et al^[5]^ suggested that cumulative 5-year survival rates for EMP fluctuate between 50% and 60%. Susnerwala et al indicated that patients with lambda light chains have a worse prognosis than patients expressing kappa light chains.^[6]^ Pratibha et al revealed that EMP prognosis does not depend on local staging. The evolution of EMP to MM is the determinant factor for survival.^[16]^ As a result, an EMP differential diagnosis should be considered in cases presenting laryngeal lesions. All laryngeal masses have to be biopsied prior to therapy to establish an accurate diagnosis, thereby leading to proper management protocols. Therefore, long-term follow-up is necessary to monitor local recurrence and the progression of MM, including local control and systemic observation via blood counts and immunoglobulin measurements. Definitive first-line treatment in EMP is based on small-scale studies due to the low incidence of EMP. Therefore, larger-scale studies are supposed to further establish and refine the current diagnosis and management guidelines, entailing major improvements in the quality of life of patients and prognosis. The specialness of our case was that the patient presented EMP masses in thyroid gland, parotid gland, larynx, the right wall of the oropharynx and nasopharynx successively, all of which were treated surgically without postoperative adjuvant radiotherapy and chemotherapy. There were no signs of progression to MM during follow-up. Unfortunately, the patient’s previous 2 surgeries were not performed in our department, and the patient’s medical history was not fully grasped. And due to insufficient understanding of the disease, the patient did not undergo bone puncture in time. When we realized that, the patient had done it in other hospital. Another special feature is the plasma that was used in this time. Low-temperature plasma radiofrequency ablation has been used in the treatment of various benign and malignant diseases of otolaryngology since 2000, but EMP resection has not been reported. We do not know what will happented to our patient, but we will continue to follow up.

## 4. Conclusion

Increasing the awareness of EMP of head and neck is warranted. Our case confirmed that surgical excision is beneficial in the treatment of small, localized EMP. Plasma is also a possible way to treat diseases.

## Author contributions

**Data curation:** Detao Ding, Xu Ma.

**Project administration:** Juxing Sun, Hui Zhang.

**Resources:** Yungang Wu.

**Writing – original draft:** Jin Zhang.

**Writing – review & editing:** YunBing Dai, Xiaoying Li, Xiaoyu Li, Yungang Wu.

## References

[1] Glasbey JC Arshad F Almond LM. Gastrointestinal manifestations of extramedullary plasmacytoma: a narrative review and illustrative case reports. Ann R Coll Surg Engl. 2018;100:371–6. 29692194 10.1308/rcsann.2018.0015 PMC5956591

[2] Burgos-Blasco B Valor-Suarez C Romo-Lopez A. Extramedullary plasmacytoma of the eyelid. J Fr Ophtalmol. 2020;43:1778. 10.1016/j.jfo.2019.07.021 31952874

[3] Alexiou C Kau RJ Dietzfelbinger H. Extramedullary plasmacytoma: tumor occurrence and therapeutic concepts. Cancer. 1999;85:2305–14. 10357398

[4] Lu G Zhang Q. Extramedullary plasmacytoma of false vocal cord: case report. Ear Nose Throat J. 2022;101:NP348–50. 33155846 10.1177/0145561320971929

[5] Kamijo T Inagi K Nakajima M. A case of extramedullary plasmacytoma of the larynx. Acta Otolaryngol. 2002;547:104–6. 10.1080/000164802760057707 12212582

[6] Pinto JA Sônego TB Artico MS. Extramedullary plasmacytoma of the larynx. Int Arch Otorhinolaryngol. 2012;16:410–3. 25991967 10.7162/S1809-97772012000300019 PMC4432537

[7] Susnerwala SS Shanks JH Banerjee SS. Extramedullary plasmacytoma of the head and neck region: clinicopathological correlation in 25 cases. Br J Cancer. 1997;75:921–7. 9062417 10.1038/bjc.1997.162 PMC2063399

[8] Jizzini MN Shah M Yeung SJ. Extramedullary plasmacytoma involving the trachea: a case report and literature review. J Emerg Med. 2019;57:e651–e67. 10.1016/j.jemermed.2019.05.032 31266689

[9] Peng L Wang R. Extramedullary intracardiac multiple myeloma misdiagnosed as a thrombus: a case report. BMC Surg. 2021;21:375–8. 34689771 10.1186/s12893-021-01377-y PMC8543969

[10] Lewis K Thomas R Grace R. Extramedullary plasmacytomas of the larynx and parapharyngeal space: imaging and pathologic features. Ear Nose Throat J. 2007;86:567–9. 17970149

[11] Ryu SW Cohen-Hallaleh V. Imaging features of extramedullary plasmacytoma. J Med Imaging Radiat Oncol. 2020;64:44–51. 31785037 10.1111/1754-9485.12975

[12] Caers J Paiva B Zamagni E. Diagnosis, treatment, and response assessment in solitary plasmacytoma: updated recommendations from a European expert panel. J Hematol Oncol. 2018;11:10. 29338789 10.1186/s13045-017-0549-1 PMC5771205

[13] Ramírez-Anguiano J Lara-Sánchez H Martínez-Baños D. Extramedullaryplasmacytoma of the larynx: a case report of subglottic localization. Case Rep Otolaryngol. 2012;2012:1–3. 10.1155/2012/437264 PMC3469077 23082263

[14] Ge S Zhu G Yi Y. Extramedullary plasmacytoma of the larynx: literature review and report of a case who subsequently developed acute myeloid leukemia. Oncol Lett. 2018;16:2995–3004. 30127889 10.3892/ol.2018.8992 PMC6096153

[15] Nakashima T Matsuda K Haruta A. Extramedullary plasmacytoma of the larynx. Auris Nasus Larynx. 2006;33:219–22. 16406428 10.1016/j.anl.2005.11.019

[16] Pratibha CB Sreenivas V Babu MK. Plasmacytoma of larynx--a case report. J Voice. 2009;23:735–8. 18619786 10.1016/j.jvoice.2008.03.009

